# Complete chloroplast genome sequence of the drought and heat-resistant Chinese alfalfa landrace, *Medicago sativa* ‘Deqin’

**DOI:** 10.1080/23802359.2019.1692715

**Published:** 2021-04-26

**Authors:** Yan Zhao, Wanyong Zeng, Wanxuan Li, Yufen Bi

**Affiliations:** a College of Horticulture and Landscape, Yunnan Agricultural University, Kunming, China; b College of Animal Science and Technology, Yunnan Agricultural University, Kunming, China; c School of Biology and Pharmaceutical Engineering, Wuhan Polytechnic University, Wuhan, China

**Keywords:** *Medicago sativa* ‘Deqin’, special landrace, chloroplast genome, phylogenetic analysis

## Abstract

*Medicago sativa* ‘Deqin’ is an excellent alfalfa landrace with strong drought and heat resistant which can grow and propagate very well in Deqin, a xerothermic valley of Jinsha River, China. In this study, the complete chloroplast genome of *M. sativa* ‘Deqin’ was assembled. The complete chloroplast genome of *M. sativa* ‘Deqin’ represents a typical circular with 125,470 bp in length, containing one inverted repeat (IR) region. Gene prediction revealed 110 genes encoding 76 proteins, 30 transfer RNAs, and four ribosome RNAs. Three genes (*rps16*, *rpl22* and *infA*) are absent. The overall GC content is 33.9%. The phylogenetic analysis revealed that *M. sativa* ‘Deqin’ belonged to the IR lacking clade, and was closely related to *M. sativa* with a 100% bootstrap support.

The genus *Medicago* contains about 87 species (Small 2010) and belongs to tribe Trifolieae, which is nested within the invert repeat lacking clad (IRLC, Cardoso et al. 2015). *Medicago sativa* L. (alfalfa) is the most important forage crop in the world. Wild type alfalfa distributed in large and continuous area as dominant species in Deqin, xerothermic valley of Jinsha River with annual precipitation of 303.9–660.0 mm, Yunnan, China (Bi et al. 2007; Ma 2010). It can grow and propagate very well with highly digestible and rich in proteins; therefore, it was used as forage by local villagers (Bi et al. 2005; Zhao et al. 2013). In 2010, it was approved by the National Grass Variety Application Committee of China as a landrace, named *M. sativa* ‘Deqin.’ However, its phylogenetic position in the genus *Medicago* is still unclear. In this study, we first reported and characterized the complete chloroplast genome of *M. sativa* ‘Deqin’ (GenBank Accession Number: MN 218692), which will be useful for its further development and utilization, and for the future phylogenetic studies of *Medicago*.

Fresh leaves of *M. sativa* ‘Deqin’ were collected from Deqin (Yunnan, China; 28°14′43.1″N, 99°18′17.9″E). Voucher specimen (BYF2004Dq003) was deposited at Herbarium, Kunming Institute of Botany, CAS (KUN). The total genomic DNA was extracted using modified CTAB method (Doyle and Doyle 1987). Reads of the complete chloroplast genome were assembled using CLC Genomic Workbench v10 (CLC Bio., Aarhus, Denmark). All the contigs were checked against the reference genome of *M. sativa* (MK460489) using BLAST (https://blast.ncbi.nlm.nih.gov/) and aligned contigs were oriented according to the reference genome. The complete chloroplast genomes were then constructed using Geneious v4.8.5 (Biomatters Ltd., Auckland, New Zealand) and was automatically annotated using DOGMA (http://dogma.ccbb.utexas.edu/). To identify the phylogenetic position of *M. sativa* ‘Deqin’, a maximum-likelihood (ML) tree was conducted by MEGA v7.0 (Kumar et al. 2016) with 1000 bootstrap replicates based on the alignments created by the online program MAFFT (https://mafft.cbrc.jp/alignment/server/index/index.html) using already published complete chloroplast genomes.

The complete chloroplast genome of *M. sativa* ‘Deqin’ represents a typical circular with 125,470 bp in length, containing only one inverted repeat region. Gene prediction revealed 110 genes encoding 76 proteins, 30tRNAs, and four rRNAs. A total of sixteen genes have one intron, and *ycf3* is the only gene with two introns. Three genes (*rps16*, *rpl22*, *infA*) are absent. The overall GC content of *M. sativa* ‘Deqin’ complete chloroplast genome is 33.9%.

To investigate the phylogenetic position of *M. sativa* ‘Deqin,’ 29 published complete chloroplast genomes of the genus *Medicago* were used to construct a phylogeny tree, using *Trigonella foenum-graceum* (MK460508) in Papilionoideae as the outgroup. The results showed that *M. sativa* ‘Deqin’ closely related to *M. sativa* with a 100% bootstrap support (Figure 1), and belonged to the IRLC which was consistent with previous study (Choi et al. 2019). This complete chloroplast genome can be subsequently used for phylogenetic and genetic engineering studies of *M. sativa* ‘Deqin,’ and would be fundamental to formulate potential development and management strategies for this special landrace.

**Figure 1.**
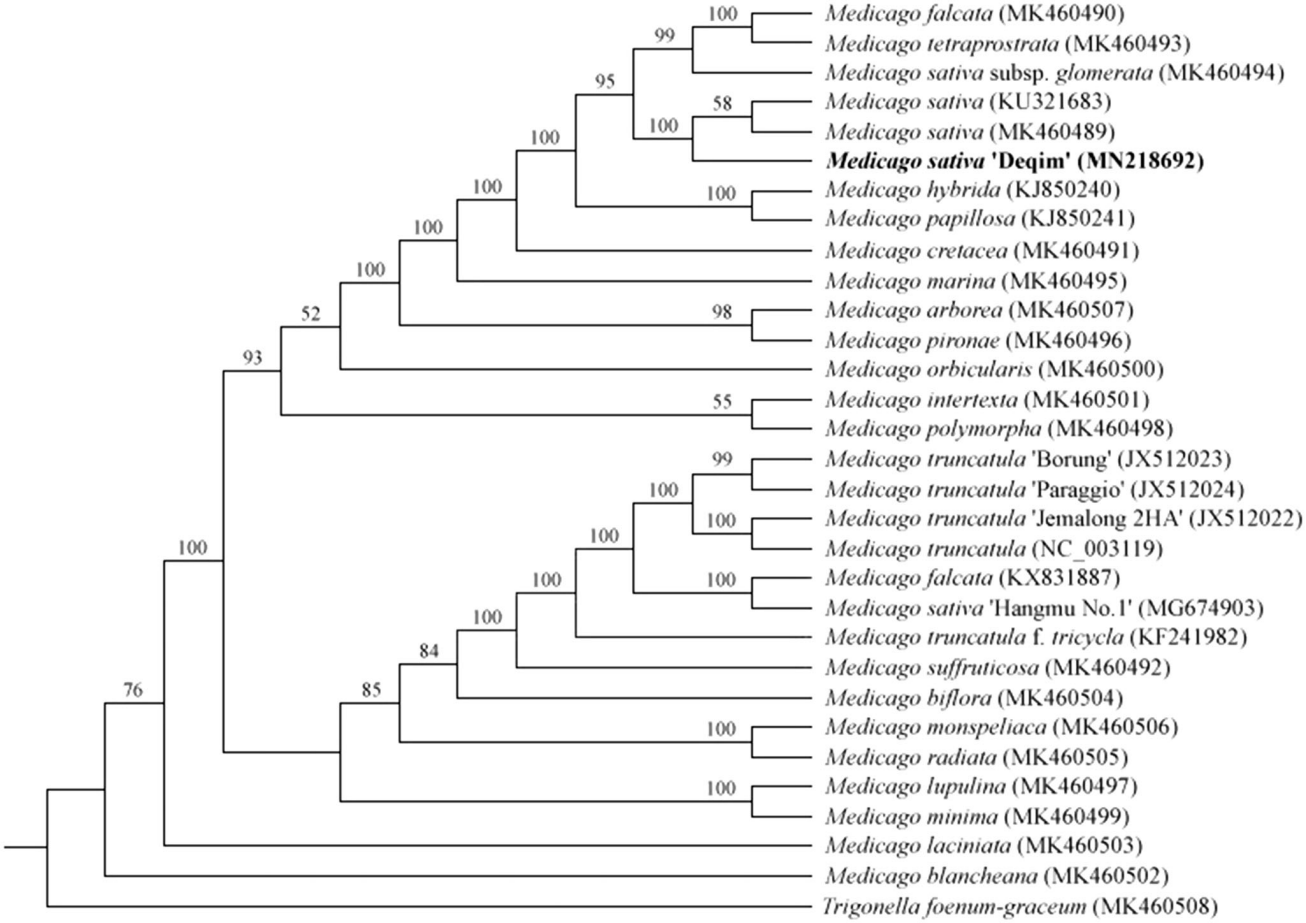
Phylogenetic relationship among *Medicago* species based on the maximum-likelihood (ML) analysis of the complete chloroplast genome sequences. Bootstrap support values (%) are indicated in each node.
